# Ureteral stricture formation after removal of proximal ureteral stone: retroperitoneal laparoscopic ureterolithotomy versus ureteroscopy with holmium: YAG laser lithotripsy

**DOI:** 10.7717/peerj.3483

**Published:** 2017-06-30

**Authors:** Henglong Hu, Lu Xu, Shaogang Wang, Xiao Yu, Huan Yang, Ejun Peng, Lei Cui, Cong Li

**Affiliations:** Department and Institute of Urology, Tongji Hospital, Tongji Medical College, Huazhong University of Science and Technology, Wuhan, People’s Republic of China

**Keywords:** Ureteral stricture, Ureteral stone, Ureteroscopy, Laparoscopic ureterolithotomy, Laser, Urolithiasis

## Abstract

**Objective:**

To compare the risk of postoperative ureteral stricture formation following retroperitoneal laparoscopic ureterolithotomy (RPLU) and ureteroscopy with holmium: YAG laser lithotripsy (URSL) in patients with proximal ureteral stones.

**Materials and Methods:**

We retrospectively reviewed the medical records of patients who underwent RPLU or URSL for proximal ureteral stones between April 2011 and May 2015. Patients were allocated into URSL group or RPLU group and the outcomes were compared.

**Results:**

A total of 201 patients who underwent 209 procedures including 159 URSL and 50 RPLU with a median follow-up of 30 months were included. No significant difference was observed among the two groups in most baseline parameters, while the stone size was significantly larger in the RPLU group (11.37 ± 2.97 vs 14.04 ± 4.38 mm, *p* = 0.000). Patients in RPLU group had markedly longer operative time (*p* = 0.000) and longer postoperative hospital stay (*p* = 0.000). The initial and one-month stone-free rates were significantly higher in the RPLU group (78.6% vs 100%, *p* = 0.000 and 82.4% vs 100%, *p* = 0.001, respectively). Patients in the RPLU had a higher complication rate (18.0% vs 9.4%, *p* = 0.098) and lower ureteral stricture rate (2.5% vs 2.0%, *p* = 1.000), while the difference was not significant. Further logistic regression model identified RPLU and female sex as independent risk factors for postoperative complication (Odds Ratio[OR] = 3.57, *p* = 0.035 and *OR* = 3.57, *p* = 0.025, respectively); however, URSL was not an independent risk factor for the formation of postoperative ureteral stricture after adjusting confounding variables (*OR* = 0.90, *p* = 0.935).

**Conclusion:**

RPLU and URSL have similar postoperative ureteral stricture formation risks. RPLU can provide significantly higher stone clearance rate, but relates with more postoperative complications.

## Introduction

Advances in minimally invasive endoscopic and laparoscopic techniques in the last few decades have remarkably changed the surgical management of ureteral stones. Nowadays, besides extracorporeal shock wave lithotripsy (ESWL), proximal ureteral stones can be managed by minimally invasive techniques like ureteroscopy, percutaneous nephrolithotomy and laparoscopic ureterolithotomy (Assimos et al., 2016; Turk et al., 2016). Although these treatment methods reach a high stone-free rate (SFR), many patients suffer from the formation of ureteral strictures after the removal of stones (Fam & Singam, 2015; Roberts et al., 1998). The prevalence of ureteral stricture in ureteral stone patients can be 3%–24% (Fam & Singam, 2015; Roberts et al., 1998). The ureteral stricture may stem from the persistent irritation of the stones and mechanical injury (Fam & Singam, 2015). Benefiting from its effectiveness for nearly all stone types, the holmium: YAG laser has been extensively used for endoscopic techniques (Turk et al., 2016). However, a recent randomized trial and meta-analysis showed that patients treated by the holmium: YAG laser have a significantly higher risk of postoperative stricture than those received pneumatic lithotripsy (Chen et al., 2017; Li et al., 2015). This was explained by the laser’s stronger ablation and coagulation effect which lead to stone fragmentation as well as ureteral thermal injury (Chen et al., 2017). As laparoscopic ureterolithotomy uses a cold knife and avoids widely intra-ureteral manipulation, it may have a lower risk of developing postoperative ureteral stricture. The primary aim of this study is to compare the risk of postoperative ureteral stricture formation in retroperitoneal laparoscopic ureterolithotomy (RPLU) and ureteroscopy with holmium: YAG laser lithotripsy (URSL).

### Patients and methods

This study has been approved by the Institutional Review Board of Tongji Hospital, Tongji Medical College, Huazhong University of Science and Technology. We retrospectively reviewed the medical records of patients who underwent surgeries for proximal ureteral stones between April 2011 and May 2015 in Tongji Hospital. The upper ureter was defined as the segment between the ureteropelvic junction and the superior margin of the sacroiliac joint. Surgical indications were calculi that failed to pass spontaneously or ESWL, recurrent renal colic pain and/or obstructive uropathy. The inclusion criteria were the presence of solitary stone in the proximal ureter and had received RPLU or URSL. Exclusion criteria included ureteral stricture diagnosed pre- or intra-operatively, pregnancy, urinary tract anatomical abnormalities, nonfunctional renal unit and loss of follow-up. Patient assessment before surgery included history taking, clinical examination, urinalysis, urine culture, complete blood count, serum biochemistry, coagulation tests, ultrasound, kidney–ureter-bladder radiography (KUB), abdominopelvic computed tomography (CT) scan and/or magnetic resonance urography (MRU). Positive urine cultures were adequately treated with appropriate antibiotics, and all patients had a negative urine culture before surgery. Stone size was determined by measuring the longest axis on preoperative imaging. In patients previously receiving anticoagulant therapy, anticoagulant was discontinued at least five days before the surgery. Patient demographics and perioperative data were recorded. The details of each procedure, and possible re-treatment, shift to other treatment, or complications had been explained to all patients before they decided on the preferred procedure. Written informed consents were taken from all patients before surgeries. Patients were allocated into URSL group or RPLU group according to the primary treatment procedure.

### Surgical procedures of URSL

Similar to previous described (Hu et al., 2016), the procedures were performed under general or epidural anesthesia with the patient in lithotomy position. In some cases, a 6 F stent had been placed one or two weeks before the procedure as a temporary measure to relieve acute obstruction, infection, or uncontrolled pain due to stones (Zhang et al., 2016). If so, the preplaced stent was removed with a rigid 8/9.8 F ureteroscope (Richard Wolf, Knittlingen, Germany) firstly. And then a guidewire was inserted into the vesicoureteric orifice and was pushed up the ureter. The rigid ureteroscope was inserted along the guidewire until it reached the level of the calculi. The stones were fragmented with a holmium laser until debris below 4 mm was achieved. Fragments were removed with a basket as many as possible. A Fr 6 double-J stent was placed at the end of the procedure routinely and was removed at one month postoperatively.

### Surgical procedures of RPLU

All patients were treated under general anesthesia in a standard kidney position. An incision of 2 cm in length was made on the middle axillary line above spina iliaca and deepened by blunt dissection until the retroperitoneal space was reached. The retroperitoneal space was widened using the index finger and a self-made gas balloon dissector. A 10 mm and a 5 mm trocar were inserted in anterior and posterior line under 12 ribs under the guide of the index finger, respectively. Carbon dioxide was used for insufflation and the pressure was kept at 10–15 mmHg. After laparoscopic instruments were introduced, the ureter segment containing stone was carefully isolated and incised to take out the stone with the ureter above clamped by separating forceps to avoid the shift of stones. The ureteral incision was closed interruptedly with 4-0 Vicryl sutures after a Fr 6 double-J stent was placed. A retroperitoneal drainage tube was indwelled and the skin incisions were closed.

### Outcome evaluation

Postoperative complications were classified according to modified Clavien system (De la Rosette et al., 2012). Initial postoperative SFR and the location of the double-J stent were evaluated on the second postoperative day with KUB. Afterwards, follow-up SFR were determined in an outpatient clinic setting at one month postoperatively with KUB or ultrasound. Ultrasound was used to assess the patients at least one-month after removing the double-J stent. When the KUB ultrasound showed light or moderate hydronephrosis without stone fragments, the patients will be recommended to recheck the ultrasound one or two month later; if the ultrasound showed severe hydronephrosis or the hydronephrosis continues grows, a computed tomography intravenous urogram (CT-IVU) or MRU was used for further assessment of the patients to confirm the formation of ureteral strictures.

### Statistical analysis

All statistical analyses were conducted using SPSS 22.0 (IBM SPSS Statistics; IBM Corporation, NY, USA) statistical software package. A Kolmogorov–Smirnov test was performed to evaluate the distributions of numeric variables. If the distribution was normal, the continuous variables was presented as mean ± standard deviation and analyzed by the Student’s t tests. However, the Mann–Whitney *U* test was used to evaluate the numerical variables with a skewed distribution which was demonstrated as median (range). Categorical variables were presented by number and frequency (%) and the Chi-square test or Fisher exact test was applied to compare proportions of categorical variables. Logistic regression analysis was performed to further assess whether postoperative ureteral stricture was associated with surgical procedures independently by adjusting potential confounding factors such as ASA grade, BMI, comorbidities, stone size, stone surgery history and hydronephrosis. Also, risk factors of short-term postoperative complication were identified in a similar way. Odds ratios (OR) and 95% confidence interval (CI) were calculated. All *p* values were 2-tailed, and *p* < 0.05 was considered as statistically significant.

## Results

Finally, 201 patients who underwent 209 procedures including 159 URSL and 50 RPLU were included into this study. Baseline demographics parameters and stone characteristics of the two groups are presented in Table 1. No significant difference was observed among the two groups in age, gender, ASA grade, BMI, comorbidities, hydronephrosis status, urine culture, preoperative stenting, stone laterality, ipsilateral stone surgery history and stone history duration, while the stone size was significantly larger in the RPLU group (11.37 ± 2.97 vs 14.04 ± 4.38 mm, *p* = 0.000).

**Table 1:** Patients and stone characteristics.

	URSL	RPLU	*p* value
Procedure number	159	50	–
Age (year)	49.21 ± 13.44	50.24 ± 12.12	0.630
Gender (male/female)	117/45	39/12	0.551
BMI	24.38 ± 3.41	23.81 ± 13.13	0.335
With/Without comorbidity	61/98	16/34	0.416
Comorbidities			0.238
Hypertension	32(18.9%)	13(26.0%)	0.378
Diabetes mellitus	13(8.2%)	4(8.0%)	1.000
Gout	7(4.4%)	0(0.0%)	0.290
Coronary heart disease	3(1.9%)	0(0.0%)	1.000
Solitary kidney	2(1.3%)	0(0.0%)	1.000
Chronic liver disease	6(3.8%)	0(0.0%)	0.364
Others	11(6.9%)	1(2.0%)	0.339
ASA grade			0.717
1	61(38.4%)	16(32.0%)	
2	84(52.8%)	29(58.0%)	
3	14(8.8%)	5(10.0%)	
Preoperative double-J stenting	3(1.9%)	0(0.0%)	1.000
Positive	6(5.4%)	4(13.8%)	
Negative	106(94.6%)	25(86.2%)	
Stone laterality (L/R)	86/73	23/27	0.318
Stone diameter (mm)	10(6–20)	14(6–25)	0.000
Hydronephrosis	145(91.2%)	46(97.9%)	0.621
Ipsilateral stone surgery history	30(37.50%)	11(22.0%)	0.684
Stone history duration			0.517
Less than 3 months	83	23	
More than 3 months	76	27	

**Notes.**

BMI body mass index L/R left/ right RPLU retroperitoneal laparoscopic ureterolithotomy URSL ureteroscopy with holmium YAG laser lithotripsy

*P* < 0.05 was considered as statistically significant.

As listed in Table 2, patients in RPLU group had markedly longer operative time (*p* = 0.000), longer postoperative hospital stay. The initial and one-month SFRs were significantly higher in the RPLU group (78.6% vs 100%, *p* = 0.000; 82.4% vs 100%, *p* = 0.001). All the short-term postoperative complications were classified as Clavien grade 1 or 2, no major complication occurred. Patients in the RPLU had a higher complication rate, while not significant. The further analysis excluding the patients with preoperative stenting generated similar results (Table S1). The logistic regression model which used to identify independent risk factors of complication revealed URSL was a significant protective factor comparing to RPLU (OR = 0.28, 95% CI [0.09–0.91], *p* = 0.035; Table 3). Also, being female was an independent risk factor for postoperative complication (OR = 3.57, 95% CI [1.17–10.87], *p* = 0.025; Table 3). In a median follow-up of 30 months for the URSL and RPLU groups, the ureteral stricture had occurred in four and one, respectively. The ureteral stricture rate was similar in the two groups (2.5% vs 2.0%, *p* = 1.000). After adjusting confounding factors by logistic regression analysis, URSL and RPLU were not significantly related with postoperative ureteral stricture formation (OR = 0.90, 95% CI [0.07–11.91], *p* = 0.935; Table 3). One patient who developed ureteral stricture in the URSL group finally received laparoscopic nephrectomy due to totally loss function of the kidney. The other three patients in URSL group and one patient in the RPLU group did not receive surgical intervention, and the hydronephrosis and renal function remained steady. During follow-up, a patient in the URSL group died of severe infection due to ureteral stone obstruction. In addition, one patient in the RPLU group developed renal failure and died of the complications.

**Table 2:** Comparisons of perioperative clinical data and outcomes.

	URSL	RPLU	*p* value
Operative time (min)	42.5(15–133)	164.5(70–330)	0.000
Short-term postoperative complication	15(9.4%)	9(18.0%)	0.098
Grade 1	5(3.1%)	5(10.0%)	0.109
Grade 2	10(6.3%)	4(8.0%)	0.992
Fever	5(3.1%)	3(6.0%)	0.620
Urine leakage	0(0.0%)	2(4.0%)	0.056
Urinary tract infection	10(6.3%)	4(8.0%)	0.992
Postoperative hospital stay(d)	4(2–10)	8(3–23)	0.000
Stenting duration (mon)	1(0.23, 6)	1(0.5, 6)	0.282
Initial SFR	125(78.6%)	50(100%)	0.000
1 month SFR	131(82.4%)	50(100%)	0.001
Long-term outcomes			
Postoperative ureteral stricture	4(2.5%)	1(2.0%)	1.000
Death	1(0.6%)	1(2.0%)	0.422

**Notes.**

RPLU retroperitoneal laparoscopic ureterolithotomy SFR stone-free rates URSL ureteroscopy with holmium YAG laser lithotripsy

*P* < 0.05 was considered as statistically significant.

**Table 3:** Results of multiple logistic regression analysis to determine factors associated with postoperative complications and ureteral stricture.

Items	Variables	Values	OR	95% CI	*p* value
Complications	Procedure	RPLU = 0; URSL = 1	0.28	0.09 − 0.91	0.035
	Sex	Male = 0; Female = 1	3.57	1.17 − 10.87	0.025
Ureteral stricture	Procedure	RPLU = 0; URSL = 1	0.90	0.07 − 11.91	0.935

**Notes.**

CI confidence interval OR odd ratio RPLU retroperitoneal laparoscopic ureterolithotomy URSL ureteroscopy with holmium: YAG laser lithotripsy

*P* < 0.05 was considered as statistically significant.

## Discussion

Ureteral calculi can cause an especially distressing condition, particularly in China, because patients often present with large and long-term impacted calculi which may be due to the lack of regular health examinations. Thanks to the advances in technology, many minimally invasive treatment procedures such as ESWL, percutaneous nephrolithotomy, ureteroscopy and laparoscopic ureterolithotomy have emerged for the treatment of ureteral stones which need surgical intervention. Although there is consensus that ureteroscopy is the most efficient treatment for patients with distal ureteral stones, there is a debate regarding large proximal ureteral stones. The American Urological Association and European Association of Urology have recommended antegrade or retrograde URSL and ESWL as first-line options, although laparoscopic ureterolithotomy may also be suitable (Assimos et al., 2016; Turk et al., 2016).

When choosing a management method, SFR is an important consideration. As expected, our results showed that the initial and one-month stone SFR were significantly higher in the RPLU group. This is also similar to the results of a recent meta-analysis which included six randomized controlled trials with 646 patients (Torricelli et al., 2016).

In addition to effectivity, safety is another consideration. RPLU has long been considered by many surgeons to be more invasive than URSL. We find no significant difference regarding perioperative complication rates in the two groups by Chi-square test. This reaffirmed the results of the meta-analysis which showed that there were no significant differences in terms of overall complications and major complications (Torricelli et al., 2016). However, after adjusting confounding factors, the logistic regression analysis found the RPLU group had a higher risk of postoperative complications.

Although the stone can be removed safely and effectively in most occasions, ureteral stricture still can be a bad legacy of it. The rates of ureteral stricture formation following endourological methods for ureteral stones were reported to be 3%∼24% (Fam & Singam, 2015; Roberts et al., 1998). Ureteral stricture patients require regular follow-up and even surgical intervention, and these surely will increase the cost. Besides the economic burden, ureteral stricture may result in hydronephrosis and life-threatening conditions such as pyonephrosis and end-stage renal failure. The formation of ureteral stricture can be explained by two reasons. One is the persistent irritation caused by stones which can result in epithelial hypertrophy, edema, fibrosis and then stricture formation, and the other is the mechanical insult of endourological methods (Fam & Singam, 2015). The first one can be reduced by the timely removal of the stones, and this is mainly based on the patients’ regular health examination. But for the second reason that issues from the treatment itself, urologists could do more, not only in improving skills but also in recommending the appropriate intervention that caused the least risk of stricture formation.

However, not many studies have been carried out to compare the occurrence of stricture formation in patients who undergo different minimally invasive treatment modality for ureteral calculi. A prospective randomized trial by Li et al., (2015) compared pneumatic lithotripsy and the holmium laser for management of middle and distal ureteral calculi and found that the use of a holmium laser was related to an increased risk of postoperative stricture. A study carried by Shao et al., (2015), they compared RPLU with URSL in the management of impacted proximal ureteral stones larger than 12 mm. Their results showed that ureteral strictures happened higher in URSL group (3.6%) than RPLU group (1.5%) with no statistical significance. Our study provided independent data on this topic and has a longer follow-up time. In addition, we identified RPLU and female sex as independent risk factors for postoperative complication, and URSL was not an independent risk factor for postoperative ureteral stricture formation after adjusting confounding variables by logistic regression model. This study would contribute to the ongoing discussion.

Compared with the RPLU, URSL has relatively more operations intra the ureter and the laser may give a thermal injury. As we tried to investigate the increased risk from the treatment method itself, we excluded patients who already had obvious ureteral stricture when receiving surgeries. The ureteral stricture formation risks were not high in both groups, and we did not find significantly higher ureteral stricture formation rate in URSL. The risk of developing ureteral stricture seems not enough to be seen as a determining factor when making a decision between the two modalities.

There are inherent limitations to the present study. First, this is a retrospective study in a single center and as such might have been subject to bias. Second, although RPLU had a higher cost than URSL in most situations, we did not compare the costs of the two procedure as these data were not available. Moreover, the limited number of patients is also the limitation of this trial. Large number randomized controlled trials are still needed to clarify this problem. However, this trial with a relatively long follow-up time could still provide the urologists and patients some valuable information in decision making and clinical consulting.

## Conclusion

RPLU and URSL are both safe methods in treating proximal ureteral stones with similar postoperative ureteral stricture formation risk. RPLU can provide a significantly higher stone clearance rate, but relates with more postoperative complications.

##  Supplemental Information

10.7717/peerj.3483/supp-1 Supplemental Information 1 Patient characteristics and outcomes

10.7717/peerj.3483/supp-2 Supplemental Information 2 Supplementary Table 1 Comparisons of perioperative clinical data and outcomes of patients without preoperative double-J stenting.

## Additional Information and Declarations

### Data Availability

The following information was supplied regarding data availability: The raw data has been supplied as a Supplementary File.
