# Supplementary material for: Ureteral stricture formation after removal of proximal ureteral stone: retroperitoneal laparoscopic ureterolithotomy versus ureteroscopy with holmium: YAG laser lithotripsy
Source: PeerJ. 2017 Jun 30;5:e3483. doi: 10.7717/peerj.3483 (PMC5494178; doi:10.7717/peerj.3483)
Supplement: Supplemental Information 2 — Comparisons of perioperative clinical data and outcomes of patients without preoperative double-J stenting. [file peerj-05-3483-s002.doc]

**Supplementary Table 1.** Comparisons of perioperative clinical data and outcomes of patients without preoperative double-J stenting.

|  | URSL | RPLU | p value |
| --- | --- | --- | --- |
| Operative time (min) | 42.5(15-133) | 164.5(70-330) | 0.000 |
| Short-term postoperative complication | 15(9.6%) | 9(18.0%) | 0.108 |
| Grade 1 | 5(3.2%) | 5(10.0%) | 0.117 |
| Grade 2 | 10(6.4%) | 4(8.0%) | 0.948 |
| Fever | 5(3.2%) | 3(6.0%) | 0.639 |
| Urine leakage | 0(0.0%) | 2(4.0%) | 0.058 |
| Urinary tract infection. | 10(6.4%) | 4(8.0%) | 0.948 |
| Postoperative hospital stay(d) | 4(2-10) | 8(3-23) | 0.000 |
| Stenting duration (mon) | 1(0.23,6) | 1(0.5,6) | 0.282 |
| Initial SFR | 122(78.2%) | 50(100%) | 0.000 |
| 1 month SFR | 128(82.1%) | 50(100%) | 0.001 |
| Long-term outcomes |  |  |  |
| Postoperative ureteral stricture | 4(2.6%) | 1(2.0%) | 1.000 |
| Death | 1(0.6%) | 1(2.0%) | 0.427 |

RPLU: retroperitoneal laparoscopic ureterolithotomy; SFR: stone-free rates; URSL: ureteroscopy with holmium: YAG laser lithotripsy.

P < 0.05 was considered as statistically significant.
